# The need for implementing a standardized, evidence-based emergency department discharge plan for optimizing adult asthma patient outcomes in the UAE, expert meeting report

**DOI:** 10.1186/s12245-024-00757-4

**Published:** 2024-11-06

**Authors:** Rasha Buhumaid, Ashraf Alzaabi, Bassam Mahboub, Mohamed Nizam Iqbal, Hamad Alhay Alhameli, Mohamed Ghazi Al-Mafrachi, Kenneth Charles Dittrich, Thiagarajan Jaiganesh

**Affiliations:** 1 Mohammed Bin Rashid University of Medicine and Health Sciences, Dubai, UAE; 2 Zayed Military Hospital, Abu Dhabi, UAE; 3 Dubai Health, Dubai, UAE; 4 Cleveland Clinic, Abu Dhabi, UAE; 5 Ibrahim Bin Hamad Obaidullah Hospital, Ras Al-Khaimah, UAE; 6 Sheikh Khalifa Medical City, Abu Dhabi, UAE; 7 Tawam Hospital, Al Ain, UAE

## Abstract

**Background:**

Asthma is a common chronic respiratory inflammatory disease that adversely affects patients’ quality of life (QoL) and overall well-being. When asthma is not adequately controlled, there is a higher risk of exacerbations and hospitalizations, thereby increasing the direct and indirect costs associated with the treatment and productivity loss. Overreliance on SABA and underutilization of ICS in the management of asthma can result in suboptimal treatment and poor asthma control. Patients who visit the emergency department are more likely to have poorly controlled asthma. Ensuring that these patients are provided with an evidence-based treatment plan during discharge can help reduce the risk of future exacerbations and consequently reduce the burden on the UAE healthcare system.

**Methods:**

A joint task force comprising experts from the Emirates Society of Emergency Medicine (ESEM) and Emirates Thoracic Society (ETS) reviewed published evidence and updated guidelines in asthma management to optimize the post-discharge recommendations.

**Results:**

The ESEM-ETS experts’ joint task force has developed a step-by-step plan for emergency department/hospital discharge, which is based on the GINA 2023 guideline recommendations and the medications available in the UAE. By adhering to this structured plan, emergency department physicians can play a crucial role in improving asthma care, long-term patient outcomes, and the utilization of healthcare resources.

**Conclusions:**

Prioritizing patient education and ensuring patients are equipped with the best-suited asthma treatment plans prior to discharge can help ED physicians improve patient outcomes and reduce healthcare resource utilization in UAE hospitals.

## Background

Asthma is a common chronic respiratory inflammatory disease in which patients present with respiratory symptoms like shortness of breath, wheezing, chest tightness, and coughing. These symptoms adversely affect patients’ quality of life (QoL), limiting their ability to engage in physical activities and sometimes even their day-to-day activities. Asthma also manifests as acute episodes or exacerbations, which may be triggered by infections, allergens, pollution, other environmental factors, and comorbidities. Exacerbations are associated with symptom worsening and can be life-threatening in some cases [1].

In 2020, the Centers for Disease Control and Prevention (CDC) reported around 716,117 asthma-related emergency department (ED) visits and 67,505 hospitalizations in the US [2]. The increased frequency of ED visits and hospitalizations contributes to the increased utilization of healthcare resources and signals inadequate asthma control in patients, a pattern also observed in the United Arab Emirates (UAE) [3, 4]. This underscores the importance of implementing nationwide strategies that can assist ED physicians in optimizing post-discharge asthma management in patients, in addition to providing acute care, with the long-term goal of reducing future ED visits.

## A call to action by the Emirates society of emergency medicine (ESEM) and Emirates thoracic society (ETS) experts’ joint task force

A joint task force comprising experts from the Emirates Society of Emergency Medicine (ESEM) and Emirates Thoracic Society (ETS) met on January 10, 2024, to discuss the current gaps in post-discharge asthma management in the UAE. The task force critically reviewed recently published evidence in asthma management and evaluated the updated national and global guidelines for asthma to endorse an evidence-based update for the National Algorithm for the Management of Adult Asthma in the ED and optimize the post-discharge recommendations. In this paper, we provide an overview of the impact of asthma on the patients and the healthcare system of the UAE and provide a rationale for implementing a standardized discharge plan across all the secondary and tertiary healthcare settings in the UAE that can help improve long-term outcomes.

## The burden of Asthma in the UAE

### Asthma treatment: goals and challenges

**Fig. 1:**
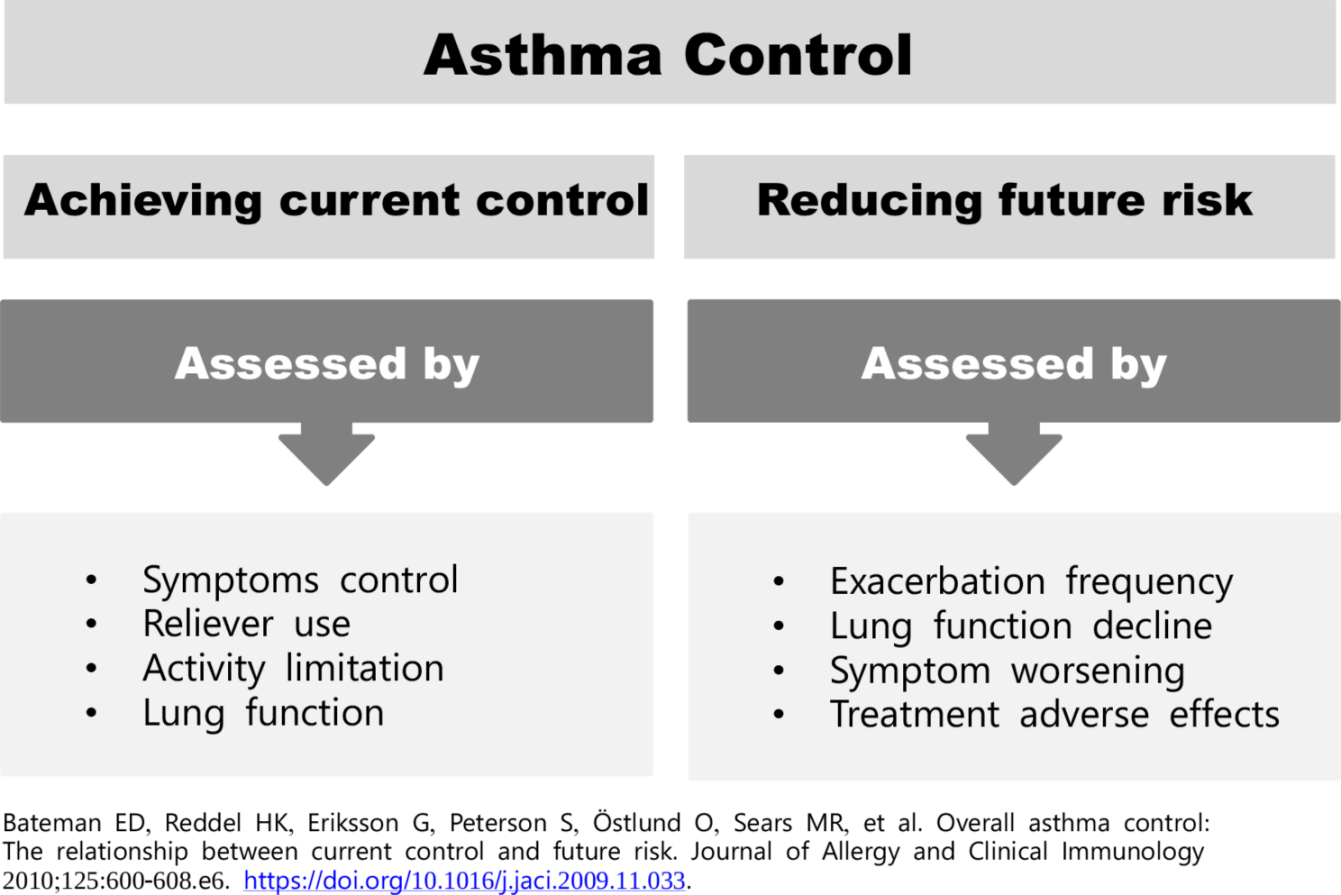
Goals of asthma management

The primary goals of asthma treatment, according to the Global Initiative for Asthma (GINA), are (1) to effectively control symptoms to ensure they do not interfere with patients’ daily activities and (2) to reduce the risk of exacerbations, death, persistent airflow limitation, and treatment side effects [5]. These goals emphasize not only achieving current symptom control but also reducing the risk of adverse outcomes in the future (see Fig. 1) [6].

Achieving these goals hinges on healthcare providers (HCPs) accurately assessing the extent of asthma control, prescribing a personalized treatment plan based on disease severity, educating patients about the importance of treatment adherence, and monitoring the level of control through regular patient follow-ups. It also depends on patients understanding the long-term goals of asthma management and the importance of future risk reduction in overall asthma control, keeping track of their symptoms, and consistently adhering to the prescribed treatments. However, a disconnect between ‘actual asthma control’ and ‘perceived asthma control has been reported in routine clinical practice. An overestimation of asthma control by patients and physicians — the perception that asthma is under control when objective assessments indicate that it is not — is a barrier to achieving asthma treatment goals. It can lead to the need for treatment escalation and cause modifiable risk factors for exacerbations, such as poor treatment adherence, to remain unaddressed [7]. Thus, despite the availability of effective treatment options that can mitigate asthma symptoms, improve long-term outcomes, and enable patients to maintain a regular and active lifestyle, sub-optimal asthma control is frequently observed in the Middle East.

### Disease burden and economic impact of Asthma

Poorly controlled asthma not only jeopardizes the achievement of long-term asthma management goals but also increases the burden on the patients and the healthcare systems. According to the Global Burden of Disease (GBD) 2019 data, the rate of age-standardized disability-adjusted life years (DALYs) globally due to asthma was 273.6 per 100,000 [8]. Epidemiological studies like SNAPSHOT and ESMAA (Assessment of Asthma Control in Adult Asthma Population in the Middle East and North Africa) have helped us gauge the burden of uncontrolled asthma on patients and the healthcare systems of the Middle East. The data from the SNAPSHOT program revealed that 38.2% of the 310 program participants from the Gulf countries had uncontrolled asthma (Asthma Control Test, ACT score ≤ 19), and significantly more participants from this cohort experienced disruptions in work/school life, social life/leisure activities, and family life/home responsibilities than those with an ACT score of > 19. SNAPSHOT results also showed that a higher percentage of participants with uncontrolled asthma sought healthcare than those with controlled asthma (72.2% vs. 27.8%) [9]. Sub-optimal asthma control was also observed in the large-scale cross-sectional ESMAA study, which included 7236 patients from 577 sites from 11 countries in the Middle East and North Africa between June 2014 and December 2015. Approximately 65% of the patients had partly controlled or uncontrolled asthma, and good adherence to prescribed treatment was seen in only 29.7% of the patients from the UAE. Moreover, > 80% of the patients had at least one risk factor for future exacerbations [10].

Thus, uncontrolled asthma is highly prevalent in the Middle East region, and it adversely affects patients’ health outcomes and QoL, leading to higher utilization of healthcare resources, including ambulatory, outpatient, and ED visits, compared to better-controlled asthma [11]. This increased resource utilization places an enormous economic burden in terms of direct (the total medical costs associated with outpatient visits, medication expenses, and hospitalizations) and indirect costs (productivity loss due to missed days of work/school) on the healthcare infrastructure in the UAE [4]. A 2019 study conducted by the public health authorities of the six GCC countries with funding from the World Bank showed that asthma resulted in productivity losses amounting to around $16.5 billion, with the UAE being the largest contributor ($7.3 billion). Moreover, the indirect costs due to asthma in the GCC countries were estimated to be nearly 22 times greater than the direct medical costs. In the UAE alone, asthma was linked to productivity losses of around $14.6 billion, with the indirect costs nearly 58 times more than the direct medical costs [12].

The burden associated with uncontrolled asthma is expected to grow continually, highlighting the need to identify and address the challenges in asthma management. In the US, it is estimated to be around $300.6 billion by the year 2039 [13].

### Challenges in asthma management

Frequent use of short-acting beta-agonists (SABAs), underutilization of inhaled corticosteroids (ICS), inadequate adherence to prescribed treatments, improper inhalation technique, and lack of health insurance have been shown to impact asthma management in the Middle East region [14]. Most of these factors are modifiable through patient or HCP-driven measures, and addressing them can improve asthma management and control and reduce the associated burden. Moreover, HCPs can reduce overdependence on SABA directly by altering their prescribing habits or indirectly by modifying patient behaviors towards prescribed ICS-based treatments. These measures can ensure good treatment adherence to ICS-based therapies, thereby improving long-term health outcomes.

A multicountry, multicenter, cross-sectional study, SABA use IN Asthma (SABINA) III, evaluated SABA use in 24 countries. In the Gulf countries of Kuwait, Oman, and the UAE, 54.5% of the 301 study participants were treated by respiratory specialists, and 58.5% were overprescribed SABA (≥ 3 canisters/year) in the previous 12 months. Moreover, 19.3% of the study participants were prescribed ≥ 10 canisters of SABA, and 13.3% had purchased it over the counter in the previous 12 months. The study found that 52.1% of the participants had either partially controlled or uncontrolled asthma, and 41.9% of the participants had experienced ≥ 1 severe exacerbation in the previous 12 months [15]. These statistics are concerning — they highlight the prevalent practices of physicians overprescribing SABA relievers as well as patients overusing them without appropriate medical oversight and are a call to action for HCPs and policymakers in the UAE to take steps to ensure that clinical practice aligns with the latest treatment recommendations.

## Continuing overreliance on SABA monotherapy: a significant hurdle in achieving asthma control

### Patient and physician factors contributing to excessive use of SABA monotherapy

Although no longer recommended as a preferred reliever in symptomatic patients, inhaled SABA has been the mainstay asthma therapy since the 1950s, providing rapid bronchodilation and quick relief from acute symptoms [16, 17]. The identification of the inflammatory component of asthma pathophysiology in the 1990s led to the recommendation of an ICS-long-acting beta-agonist (LABA) combination as maintenance therapy and prompted switching inhaled SABAs to an as-needed treatment modality in the early 2000s [16]. Despite the 2019 GINA paradigm-shifting recommendation discouraging the use of SABA-only relievers, persistent reliance on it continues in the Gulf Region due to multiple physician- and patient-related factors, posing a challenge in achieving optimal asthma control (see Table 1) [15, 17–20]. As a part of the international CARBON program, a study retrospectively assessing the carbon footprint of SABA and controller inhalers across various countries found that SABA utilization accounted for 70% of total inhaler use and SABA-related greenhouse gas (GHG) emissions constituted 78% of the total inhaler-related GHG emissions in the UAE [21].

**Table 1:** Patient and physician factors that lead to overreliance on SABA-only relievers

Patient behaviors and perceptions that lead to overreliance on SABA-only relievers	Physician factors that contribute to overprescribing of SABA-only relievers	Other factors
• Failure to understand that although SABA monotherapy might provide immediate symptom relief, it is associated with adverse outcomes in the long term as it does not treat the underlying inflammation • Reluctance to take the prescribed maintenance treatment during symptom-free periods • Cultural norms of “medicine sharing” and “doctor shopping,” which are common in the Gulf countries • Unwarranted fear of treatment side-effects	• Prioritizing patient preferences over adherence to clinical guidelines • Insufficient asthma management training • Therapeutic decisions influenced by treatment cost	• The dynamics of the doctor-patient relationship • High cost of maintenance treatment • Insurance coverage • Accessibility to medications

Alzaabi A, Al Busaidi N, Pradhan R, Shandy F, Ibrahim N, Ashtar M, et al. Over-prescription of short-acting β2-agonists and asthma management in the Gulf region: a multicountry observational study. Asthma Res Pract 2022;8:3. 10.1186/s40733-022-00085-5

Zhang X, Quint J. Online Survey to Investigate Asthma Medication Prescription and Adherence from the Perspective of Patients and Healthcare Practitioners in England. J Asthma Allergy 2023;Volume 16:987–96. 10.2147/JAA.S426227

Kaplan A, Mitchell PD, Cave AJ, Gagnon R, Foran V, Ellis AK. Effective Asthma Management: Is It Time to Let the AIR out of SABA? J Clin Med 2020;9:921. 10.3390/jcm9040921

Shayo GA, Omary A, Mugusi F. Inhaler Non-Adherence, Associated Factors and Asthma Control among Asthma Patients in a Tertiary Level Hospital in Tanzania. East African Health Research Journal 2022;6:78–85. 10.24248/eahrj.v6i1.682

Domingo C, Singh D. The Changing Asthma Management Landscape and Need for Appropriate SABA Prescription. Adv Ther 2023;40:1301–16. 10.1007/s12325-022-02410-z

Asthma patients have become so accustomed to SABA relievers that they instinctively reach for them whenever symptoms arise, often without realizing that the underlying inflammation is not being addressed. Thus, to reduce patients’ excessive reliance on as-needed SABA relievers, GINA introduced the concept of the ‘anti-inflammatory reliever’ (AIR). The AIR involves the use of a reliever inhaler that includes a low-dose ICS and a fast-acting bronchodilator, i.e., ICS-formoterol for Track 1 patients or ICS-SABA for Track 2 patients. While the SABA-only reliever provides instant symptom relief, the rationale behind AIR use is to provide rapid symptom relief and reduce the risk of exacerbations in the future due to the inclusion of a small dose of ICS [5, 20].

Inadequate anti-inflammatory treatment can cause inflammation to go unchecked, decreasing lung function, worsening asthma control, increasing exacerbation risk, and causing airway remodeling [16, 22]. Regular SABA use can increase bronchial hyper-responsiveness, thereby making patients more sensitive to asthma triggers [23]. Overuse of SABA has been shown to increase the incidence of ED visits, hospitalizations, and the use of oral corticosteroids, and in an ED setting has also been associated with transient lactic acidosis, tachycardia, arrhythmias, QTc interval prolongation, hypokalemia, hypomagnesemia, muscle tremors and cramps, and anxiety [14, 24]. Additionally, overreliance on SABA relievers, which signals poor asthma control, has also been linked to an increased risk of death, prompting the Royal College of Physicians to recommend an urgent review of all patients prescribed > 12 short-acting relievers in the previous 12 months [25].

The US administrative claims data from IBM MarketScan research databases were analyzed to correlate the real-world prescription fill trends of SABA and maintenance medications with severe exacerbations in asthma patients (≥ 12 years old, *N* = 135,540). The results showed that 48.5% of the patients categorized as having well-controlled asthma (defined by 1 SABA fill per year) and reporting 53% adherence to maintenance medication also had at least one severe exacerbation per year. The findings indicate that there are gaps in the real-world SABA and maintenance therapy usage trends. The real-world use of SABA and maintenance therapy may not fully address the fluctuating nature of airway inflammation associated with asthma or eliminate the risk of asthma exacerbations, highlighting the importance of filling these gaps [26].

To reduce the risk of inappropriate use and overreliance on SABA-only therapy and to ensure inflammation is effectively targeted early on, experts from the Middle East and Africa (MEA) region and key opinion leaders (KOLs) from the Gulf Region have also advocated the adoption of SABA-alone-free clinical practice in both outpatient and ED settings and recommended the use of the ICS-SABA combination replacing SABA monotherapy in GINA Track 2 patients and facilitation of its availability in the region [14, 27]. The availability of both the ICS and SABA components in a single inhaler device can help reduce the risk of non-adherence associated with using 2 separate inhaler devices [27]. Additionally, the combination, which allows concomitant treatment of symptoms and inflammation, might ensure this ‘window of opportunity’ to reduce future exacerbation risk is not lost [28].

Patient behavior that tends to downplay the need for daily maintenance treatment and prioritize quick relief with a SABA inhaler represents a significant barrier to achieving optimal asthma control. The International Asthma Patient Insight Research (INSPIRE) study evaluated the attitudes towards asthma management of 3415 patients (≥ 16 years) from 11 countries with physician-confirmed asthma diagnoses and who were prescribed regular maintenance ICS therapy (with or without LABA). The study showed that 74% of these patients relied on at least one inhalation of SABA daily. Also, the level of control perceived by the patients varied from that assessed by the Asthma Control Questionnaire (ACQ). The study found that 87% of patients evaluated by the ACQ as having ‘not well-controlled’ asthma perceived their asthma control as ‘relatively good.’ Moreover, 55% of those with ‘uncontrolled asthma’ also thought their asthma control was ‘relatively good.’ Additionally, 54% of the patients felt concerned about taking medications during symptom-free periods, 39% believed there was no need to take daily treatment when they felt well, and 90% preferred treatments providing instant relief [29]. Patient misconceptions about the importance of maintenance treatment and overreliance on SABA increase the risk of non-adherence to prescribed treatments and underscore the pivotal role of patient education in overcoming this barrier.

### The role of ED physicians in asthma care

We believe patients presenting to the ED with exacerbations represent a cohort with poorly controlled asthma, providing the ED physicians in the UAE hospitals with a unique opportunity to intervene favorably to optimize the disease course proactively. The role of ED physicians must extend beyond treating the acute symptoms to ensuring that patients are discharged on the best-suited, evidence-based asthma treatment plans. The ED physicians should be encouraged to identify the causes of exacerbations and take steps to help reduce the exacerbation frequency and, consequently, future ED visits. They can help patients identify and avoid triggers while emphasizing the importance of treatment adherence and guiding them on using the inhaler correctly, including with spacers when necessary. Educating patients on identifying triggers and warning signs of an impending exacerbation can allow them to seek early intervention. ED physicians can train patients to use peak flow meters and encourage them to maintain a diary with readings. Additionally, they can encourage patients to follow up with their primary care physicians for mild cases and with specialist pulmonologists for moderate-to-severe cases, who can evaluate the use of advanced therapies such as immunotherapy.

Collectively, these steps can help optimize long-term asthma control, reduce the burden on the UAE healthcare system, and contribute to a more efficient and sustainable healthcare framework.

### Beyond the ED: optimizing a standardized discharge plan for adult asthma patients

**Fig. 2:**
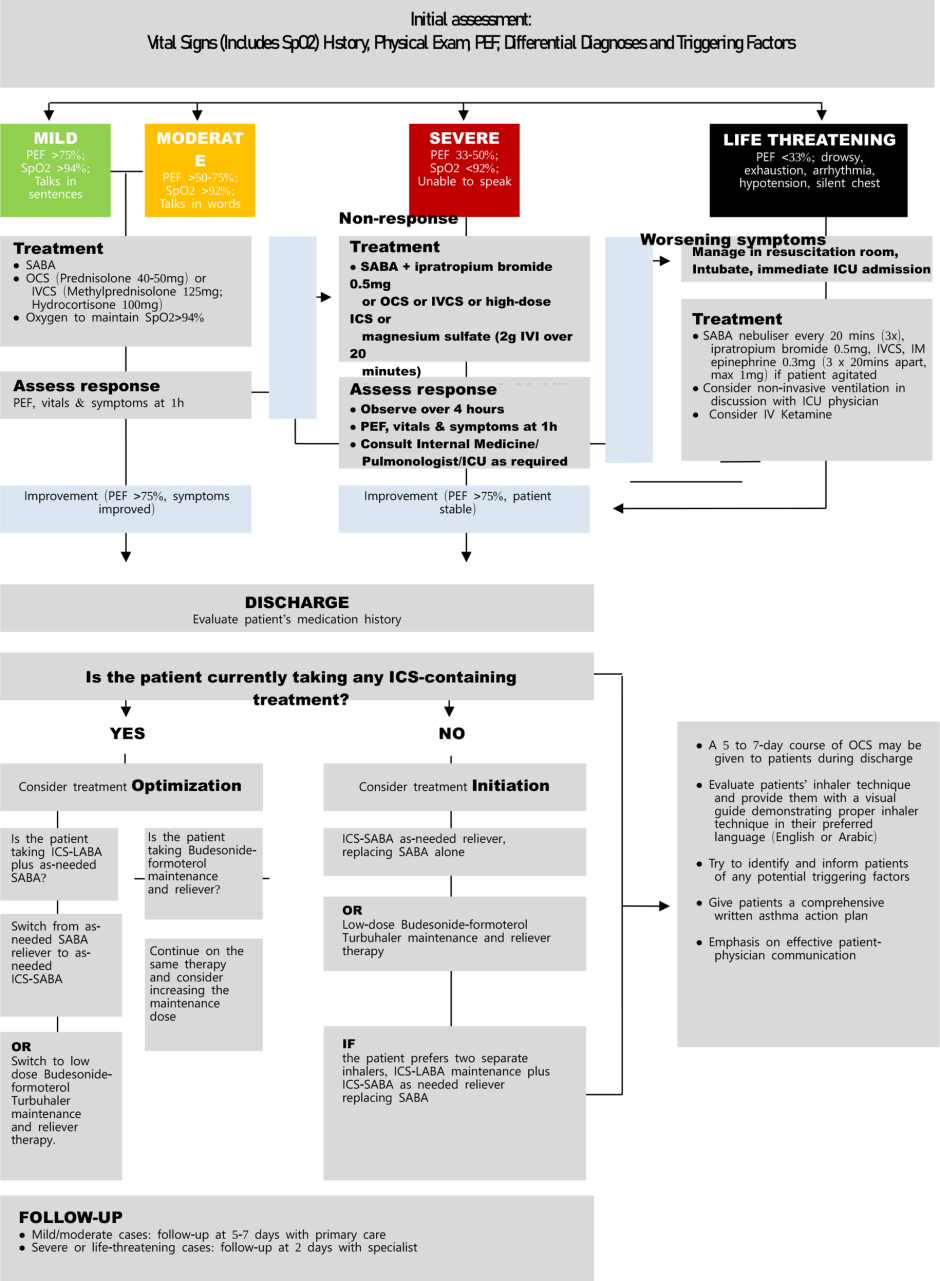
Discharge plan integrated into the National algorithm for the management of adult asthma in the ED

The ESEM-ETS experts’ joint task force proposes establishing a structured, straightforward, and easy-to-follow discharge plan comprising a post-discharge asthma management strategy that can be integrated seamlessly into the UAE National Algorithm for the Management of Adult Asthma in the ED (see Fig. 2). This discharge plan aligns with the most recent GINA guidelines and can be used across all the hospitals in the UAE. The benefits of such a discharge plan encompassing a post-discharge management strategy will be two-fold — (1) It will allow all ED physicians to consistently adhere to GINA guidelines and practice evidence-based care, and (2) It will also help patients receive guidance to manage their symptoms optimally after getting discharged from a hospital. The availability of a discharge plan can streamline the discharge process without putting additional undue burden on busy ED physicians. The goals of implementing this discharge plan are shown in Table 2.

**Table 2:** Goals of implementing a standardized discharge plan in the UAE hospitals

• To ensure consistent, evidence-based care	
• To improve patient outcomes by reducing the frequency of exacerbations and ED visits	
• To promote efficient physician-patient communication	
• To optimize resource utilization	
• To reduce the disease burden on patients and the strain on healthcare resources	

The joint task force also proposes implementing specific Key Performance Indicators (KPIs) that could be used to objectively evaluate treatment outcomes following patients’ index exacerbation (see Table 3). These KPIs would provide insights into the effectiveness of ED physicians’ prescribed interventions and help identify areas for further optimizing post-discharge patient care.

**Table 3:** Proposed key performance indicators (KPIs) during the measurement year post-index exacerbation

KPI	Measure target
Percentage of adult patients and adolescent patients with acute asthma exacerbation discharged with an Anti-Inflammatory Reliever (AIR), either ICS-SABA or ICS-formoterol during the measurement year.	50% or higher of the total discharged patients.
Percentage reduction in asthma exacerbations requiring emergency department visits during the measurement year.	Higher is better
Percentage reduction in average use of short-acting beta-2 agonist (SABA) reliever in post- discharge patients during the measurement year.	40% or higher of the total discharged patients.

## Discussion

Suboptimally controlled asthma imposes a substantial burden on the patients and the healthcare system of the UAE. From a patient’s perspective, it leads to impaired QoL and limits daily activities. It is also associated with an increased risk of exacerbations and hospitalizations [14, 30]. The higher the frequency of exacerbations and hospitalizations, the higher the direct and indirect costs related to medical care and productivity losses, including treatment costs, hospital stays, absenteeism, and decreased work efficiency [15]. Familiarity with SABA relievers, which provide quick relief from symptoms, has led to asthma patients relying excessively on them. Overreliance on SABA and underutilization of ICS due to patient- or physician-related factors can result in suboptimal treatment of underlying inflammation and, consequently, poor asthma control. Identifying patients with poorly controlled asthma, understanding the contributing factors, and intervening early to mitigate the risk of future exacerbations is crucial to reducing the burden on the UAE healthcare system and improving long-term patient outcomes.

Patients presenting to the ED, especially those with mild to moderate asthma, are more likely to have poorly controlled asthma and represent a population with unmet healthcare needs. Apart from treating their acute symptoms, ED physicians can play a crucial role in helping these patients achieve the long-term asthma treatment goal of future exacerbation risk reduction by identifying potential barriers to asthma management, optimizing treatment plans using evidence-based strategies, explaining the difference between perceived and actual asthma control, and educating them on the importance of adhering to prescribed treatments.

The ESEM-ETS experts’ joint task force has formulated a step-by-step ED/hospital discharge plan based on the GINA 2023 guideline recommendations and the availability of medications in the UAE. Implementing this discharge plan across hospitals can increase efficiency in patient care, enhance patient-ED physician communication, and facilitate continuity of treatment post-discharge from the ED. Guidance provided to patients for ongoing asthma management during the discharge process can help improve their overall asthma control and QoL. Moreover, a follow-up study can be conducted to understand how implementing this easy-to-follow discharge plan impacts real-world clinical practice, patient outcomes regarding ER visit frequency and hospitalizations, and healthcare costs in the UAE.

## Conclusions

Implementing the discharge plan proposed by the ESEM-ETS experts’ joint task force in hospital EDs has the potential to significantly improve post-discharge asthma control in patients, thereby reducing the exacerbation frequency. By prioritizing patient education and ensuring patients are equipped with the best-suited asthma treatment plans prior to discharge, ED physicians can help improve patient outcomes and reduce healthcare resource utilization in UAE hospitals.

## Data Availability

No datasets were generated or analysed during the current study.
